# Unveiling Cardiovascular Risk Among Older Adults in Eastern India: A Community-Based Cross-Sectional Study

**DOI:** 10.7759/cureus.85063

**Published:** 2025-05-29

**Authors:** Bijit Biswas, G. Jahnavi, Hem Nandani Pathak, Anuradha Gautam, Richa Richa, Arshad Ayub, Pratima Gupta, Saurabh Varshney, Sudip Bhattacharya, Sunil Kumar Panigrahi, Rajesh Kumar

**Affiliations:** 1 Community and Family Medicine, All India Institute of Medical Sciences, Deoghar, IND; 2 Microbiology, All India Institute of Medical Sciences, Deoghar, IND; 3 Otolaryngology, All India Institute of Medical Sciences, Deoghar, IND; 4 General Medicine, All India Institute of Medical Sciences, Deoghar, IND

**Keywords:** adult, cardiovascular diseases, risk, rural population, urban population

## Abstract

Background

Cardiovascular disease (CVD) poses major public health challenges in low-resource settings like India, where it contributes significantly to premature mortality and morbidity. This study assessed 10-year CVD risk and its associated factors among community-dwelling older adults in Eastern India.

Methods

This cross-sectional study, conducted in rural and urban areas of Deoghar, Jharkhand, in 2023, assessed 477 adults (aged 40-74 years) using the World Health Organization/International Society of Hypertension (WHO/ISH) South Asian Region (SAR) non-laboratory risk chart. Multinomial logistic regression identified predictors of moderate-to-high CVD risk.

Results

Among participants, 75.8% had a low 10-year CVD risk (< 10%), 22.2% had moderate risk (10% to <20%), and 1.9% had high risk (≥20%). Predictors of moderate-to-high CVD risk (≥10%) identified through multinomial logistic regression included increasing age (adjusted odds ratio (AOR): 2.0; 95% confidence interval (CI): 1.8-2.3), male gender (AOR: 16.0; 2.4-106.3), lower per capita monthly income (PCMI) (AOR: 3.0; 1.0-8.9), family history of hypertension, diabetes, or heart disease (AOR: 5.7; 1.8-18.4), central obesity (AOR: 11.9; 3.5-40.9), and tobacco use (AOR: 8.2; 2.0-33.6). Regular physical activity (≥30 minutes/day) was a protective factor (AOR: 0.2; 0.1-0.8). The model accounted for 81.8% of the variability in cardiovascular risk outcomes.

Conclusions

About one-fourth of older adults were identified as having moderate-to-high 10-year CVD risk. Central obesity and tobacco use emerged as significant predictors, while regular physical activity offered protective benefits. Implementing targeted interventions to address modifiable risk factors is the need of the hour to mitigate CVD risk.

## Introduction

Cardiovascular diseases (CVDs) have long been the leading cause of death globally, accounting for 20.5 million deaths in 2021, or about one-third of all global fatalities. This represents a sharp increase from 12.1 million CVD-related deaths in 1990 [1,2]. Ischemic heart disease (IHD) is now the leading cause of premature death, particularly in South Asia, with India bearing a significant portion of this burden. In India, CVDs are responsible for 26.6% of all deaths and 13.6% of disability-adjusted life years (DALYs), with a 2.3-fold rise in the prevalence of IHD and stroke between 1990 and 2016 [3-5]. The age-standardized death rate for CVD in India (282 per 100,000 people) far exceeds the global average of 233, making CVD a critical public health challenge [4].

The increasing prevalence of CVDs in India is primarily driven by modifiable risk factors such as hypertension (21.3% women to 24.0% men), tobacco use (8.9% women to 38.0% men), poor diet (98.0% men to 98.8% women), obesity (22.9% men to 24.0% women), physical inactivity (30.9% men to 52.4% women), elevated blood sugar (13.5% women to 16.5% men), and hypercholesterolemia (ranging regionally from 4.6% to 50.3%) [5-8]. These risk factors are more widespread in India than in high-income countries, exacerbating the situation. The economic impact of CVDs is substantial, with healthcare costs estimated at $7.5 billion in 2010, pushing many households into poverty [4,5,9,10]. Managing cardiovascular diseases in hospitals is also costly, with per-patient expenses reaching INR 2,25,293 (USD 3,476), which rises to INR 2,47,822 (USD 3,824) when accounting for administrative overheads [11]. While addressing these modifiable risk factors could significantly reduce the burden of CVDs, implementation of effective interventions remains limited, particularly in resource-constrained settings [4,5,9,12].

In such settings, tools like the World Health Organization (WHO) and International Society of Hypertension (ISH) non-laboratory-based CVD risk prediction charts offer a cost-effective solution for identifying high-risk individuals. These charts assess simple parameters such as age, gender, smoking status, systolic blood pressure, and body mass index (BMI), enabling timely interventions that can avert catastrophic healthcare costs [9,12]. Despite the potential of these tools, studies applying them at the population level in India, especially in the eastern region, remain limited [13-16]. This study aims to address this gap by assessing the 10-year CVD risk and its predictors among community-dwelling older adults in Eastern India. The findings will provide valuable insights to inform targeted interventions, aiming to reduce the rising burden of CVDs in the region.

## Materials and methods

Study design and setting

This cross-sectional study was conducted between January and December 2023 in urban and rural outreach areas under the Department of Community and Family Medicine, All India Institute of Medical Sciences (AIIMS) Deoghar, located in Jharkhand, India. It was integrated with the community-based teaching curriculum for undergraduate medical students, including Clinico-Psycho-Social Case Reviews (CPSCR) and comprehensive family health assessments.

Sample size and procedure

**Figure 1:**
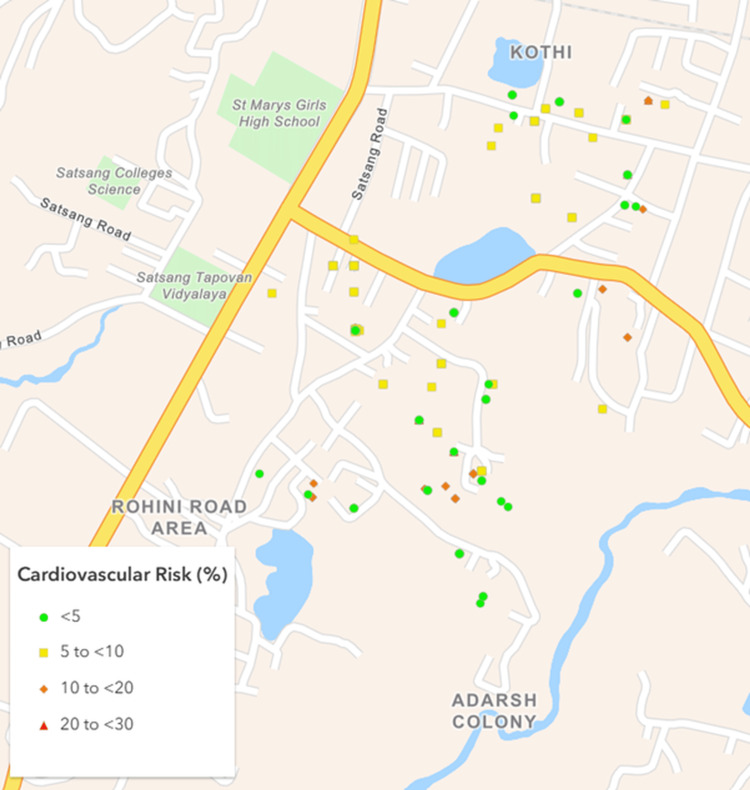
Map of the urban cluster showing geographical distribution of the study participants as per their cardiovascular risk. Created using ArcGIS Online software (Esri, Redlands, USA).

**Figure 2:**
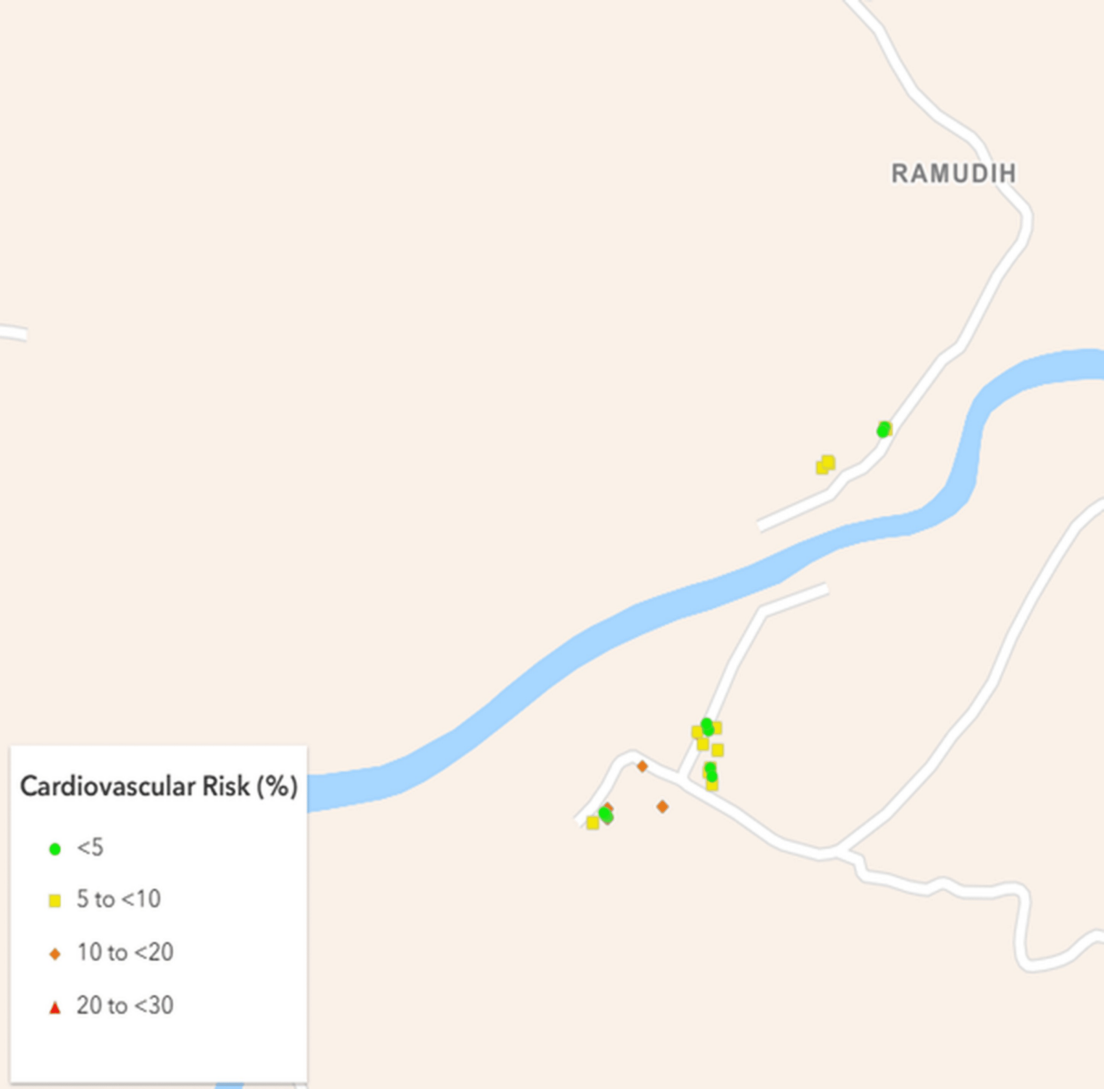
Map of the rural cluster I showing geographical distribution of the study participants as per their cardiovascular risk. Created using ArcGIS Online software (Esri, Redlands, USA).

**Figure 3:**
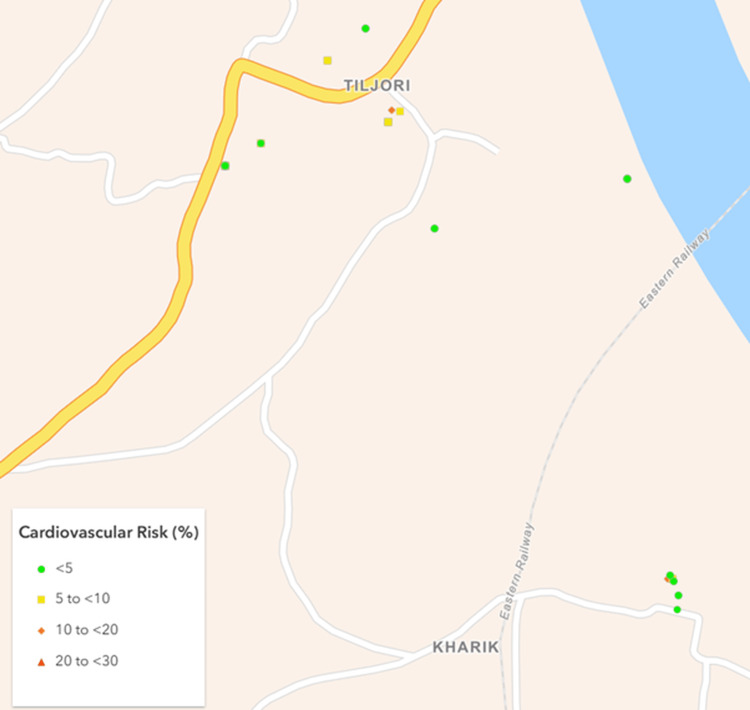
Map of the rural cluster II showing geographical distribution of the study participants as per their cardiovascular risk. Created using ArcGIS Online software (Esri, Redlands, USA).

**Figure 4:**
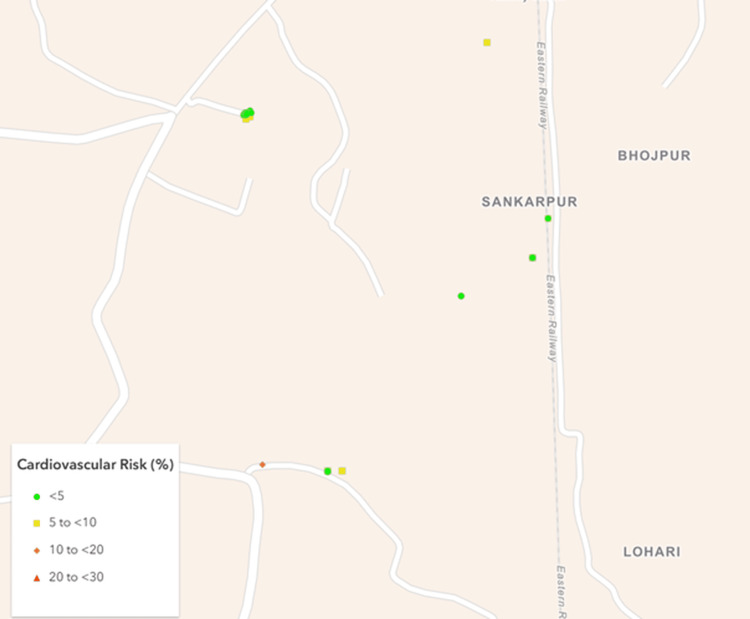
Map of the rural cluster III showing geographical distribution of the study participants as per their cardiovascular risk. Created using ArcGIS Online software (Esri, Redlands, USA).

Based on a nationally representative study by Kulothungan et al. [17], it was assumed that at least 15% of participants would have a ≥10% 10-year cardiovascular risk. Using a 5% absolute precision and a design effect of 2, the minimum sample size was calculated as 392 using Statulator, an online sample size calculator [18]. Ultimately, 477 participants were successfully recruited from eight villages and five urban mohallas (neighborhoods) (Figures 1-4).

In the CPSCR program, students handled cases involving under-five children, geriatric patients, individuals with diabetes or hypertension, antenatal and postnatal care, and tuberculosis, alongside family diagnoses. They gathered data on sociodemographic and socioeconomic profiles, housing and environmental conditions, socio-cultural factors, nutritional status, and preventive health practices. Students also identified health determinants, formulated a clinico-psycho-social family diagnosis, and provided tailored recommendations. During clinical postings, all adults aged 40 to 74 years from families assigned to students were invited to participate in the study. Those who consented underwent data collection by the investigators, who recorded anthropometric measurements, blood pressure (BP), and blood glucose levels. Participants with elevated BP or blood glucose were managed per standard treatment guidelines [19,20] and referred to nearby healthcare facilities if necessary. Disease-specific self-care advice was also provided, and participants were thanked for their involvement at the study's conclusion.

Operational definitions

Socioeconomic status: It was assessed based on possession of a Below Poverty Line (BPL) red ration card, issued according to government-defined criteria. Individuals without a BPL red ration card were classified as Above Poverty Line (APL) [21].

Tobacco Use: Use of smoked or smokeless tobacco in the past 30 days [22,23].

Alcohol Use: Consumption of alcohol in the past 30 days [22,23].

Fruit and Vegetable Intake: Adequate intake is defined as consuming ≥5 servings per day [22,23].

Physically Active: Engaging in at least 30 minutes of moderate to vigorous physical activity daily [23].

Waist Circumference (WC): Measured at the midpoint between the lowest rib and iliac crest at the end of expiration using a non-stretchable tape to the nearest 0.1 cm [22,24].

Centrally Obese: WC ≥90 cm for men and ≥80 cm for women [22,24].

Hip Circumference (HC): Measured at the widest part of the buttocks using a non-stretchable tape [22,24].

High Waist-Hip Ratio: Defined as ≥0.90 for men and ≥0.85 for women [22,24].

Height: Measured with participants standing upright, heels against the wall, feet together, and breathing normally using a non-stretchable tape to the nearest 0.1 cm after removing footwear and headgear [22,24].

Body Weight: Measured using a calibrated scale with participants wearing light clothing and no footwear [22,23].

Overweight and Obesity: Defined as Body Mass Index (BMI) ≥23 kg/m² and ≥25 kg/m², respectively [22].

Blood Pressure (BP): Measured on the right arm in a seated position using a digital sphygmomanometer. Two readings were taken one minute apart, and the mean was recorded [22].

Hypertension: Defined as systolic BP ≥140 mmHg, diastolic BP ≥90 mmHg, or current use of antihypertensive medication [22].

Blood Glucose: Random blood glucose was measured using a digital glucometer [22].

Diabetes: Defined as blood glucose ≥200 mg/dL or current use of diabetes medication [22].

Cardiovascular Risk Assessment: The 10-year cardiovascular risk was assessed using the WHO/ISH non-laboratory-based risk prediction chart for the South Asian Region. The parameters considered were age, gender, systolic blood pressure (SBP), smoking status, and body mass index (BMI). Risk levels were categorized into five groups: <5% (green), 5% to <10% (yellow), 10% to <20% (orange), 20% to <30% (red), and ≥30% (deep red) [12].

Data collection and analysis

Data were collected using Epicollect 5 [25], an open-source platform, and exported to Excel (Microsoft Corporation, Redmond, USA) for analysis in JAMOVI (version 2.3.28) [26]. Quantitative variables were summarized as mean (standard deviation, SD) or median (interquartile range, IQR), depending on their distribution. Qualitative variables were presented as frequency (percentage) with 95% confidence intervals (CI). Associations between background characteristics and 10-year cardiovascular risk were analysed using the chi-square test. Bivariate multinomial logistic regression was conducted to identify associated factors, followed by multivariable multinomial logistic regression for further analysis. Variables with a p-value <0.250 in the bivariate analysis were included in the multivariable model. Results were reported as odds ratios (OR) with 95% CI, and a p-value <0.05 was considered statistically significant. The maps were created using ArcGIS Online software (Esri, Redlands, USA).

The study received approval from the Institutional Research Committee (IRC) and Institutional Ethics Committee (IEC) of AIIMS Deoghar (Ref No. 2022-79-IND-02).

## Results

Most of the study participants were females, 300 (62.9%), and under 60 years of age 311 (65.2%), with an age range of 40 to 72 years. The majority were Hindu by religion 454 (95.2%) and illiterate 258 (54.1%). About 221 (46.3%) were homemakers, and 220 (46.1%) lived in urban areas. Approximately 39 (8.2%) were underweight, while 85 (17.8%) were overweight and 136 (28.5%) were obese. About 266 (55.8%) had central obesity, and 356 (74.6%) had a high waist-hip ratio. Only 92 (19.3%) engaged in at least 30 minutes of moderate to vigorous physical activity, while 299 (62.7%) did not engage in physical activity at all. Overall, 143 (30.0%) consumed tobacco, with the majority being smokeless tobacco users 119 (24.9%), followed by smokers 15 (3.1%), and dual users nine (1.9%). *Khaini *was the most commonly used tobacco product 115 (24.1%), followed by *gutka *23 (4.8%), *gul *19 (4.0%), *biri *17 (3.6%), and cigarettes 13 (2.7%) (*khaini *is a form of smokeless tobacco made of sun-dried tobacco leaves mixed with slaked lime; *gutka *is a commercially prepared mixture of areca nut, tobacco, and flavoring agents; *gul *is a powdered tobacco preparation used for dental cleaning; and *biri *(*beedi*) is a traditional hand-rolled tobacco cigarette made using tendu leaves). Among the participants, 51 (10.7%) were alcohol users, with 49 (10.3%) preferring country liquor and two (0.4%) preferring foreign liquor. The majority 330 (69.2%) consumed at least 5 grams of salt per day, while 169 (35.4%) added salt to their food and 136 (28.5%) consumed salt-rich foods. Regarding oil consumption, about 276 (57.9%) consumed at least 500 millilitres of oil per month, while only 61 (12.8%) consumed five or more servings of fruits and vegetables. In terms of comorbidities, 202 (42.3%) were hypertensive, 95 (19.9%) were diabetic, and 46 (9.6%) had both hypertension and diabetes (Table 1).

**Table 1:** Background Characteristics of the Study Participants: (n=477) APL: above poverty line, BMI: body mass index,  BPL: below poverty line, CI: confidence interval, cm: centimetre. IQR: interquartile range, kg: kilogram, m: meter, min: minutes, OBC: other backward caste, PCMI: per capita monthly income, SC: scheduled caste, SD: standard deviation, ST: scheduled tribe, USD: United States Dollar, Professional: Doctor (2), Advocate (2); Semi-professional: High school teacher (6); Skilled: Shopkeeper (22), Farmer (51), Driver (9), Electrician (12), Mason (4), Barber (4), Cook (1), Health Worker (25); Semiskilled:  Labourer (39), Hawker (2); Unskilled: Watchman (2), Domestic Servant (6).

Characteristics	Values
Sociodemographics	
Age, y, mean ± SD	53.9 ± 9.4
Gender, n (%;(95% CI))	
Male	177 (37.1; 32.8-41.5)
Female	300 (62.9; 58.4-67.1)
Religion, n (%;(95% CI))	
Hindu	454 (95.2; 92.9-96.8)
Muslim	23 (4.8; 3.2-7.1)
Caste, n (%;(95% CI))	
OBC	203 (42.6; 38.2-47.0)
SC	106 (22.2; 18.7-26.2)
ST	61 (12.8; 10.1-16.1)
Others	107 (22.4; 18.9-26.4)
Educational Level, n (%;(95% CI))	
Illiterate	258 (54.1; 49.6-58.5)
Below Primary	32 (6.7; 4.8-9.3)
Primary	58 (12.2; 9.5-15.4)
Middle	30 (6.3; 4.4-8.8)
Secondary and above	99 (20.8; 17.4-24.6)
Place of residence, n (%;(95% CI))	
Rural	257 (53.9; 49.4-58.3)
Urban	220 (46.1; 41.7-50.6)
Socioeconomic	
Socioeconomic status, n (%;(95% CI))	
APL	365 (76.5; 72.5-80.1)
BPL	112 (23.5; 19.9-27.5)
Occupation, n (%;(95% CI))	
Skilled	128 (26.8; 23.1-30.9)
Semiskilled	41 (8.6; 6.4-11.4)
Unskilled	8 (1.7; 0.8-3.3)
Semi-professional	6 (1.3; 0.5-2.7)
Professional	4 (0.8; 0.3-2.1)
Homemaker	221 (46.3; 41.9-50.8)
Unemployed	8 (1.7; 0.8-3.3)
At home	61 (12.8; 10.1-16.1)
Per capita monthly income (PCMI), USD, median (IQR)	34.9 (21.8, 49.9)
Family History	
Family history of high blood pressure, diabetes and heart disease, n (%;(95% CI))	121 (25.4; 21.7-29.5)
Measurement	
BMI, kg/m^2^, mean ± SD	23.3 ± 4.0
Waist circumference, cm, mean ± SD	81.8 ± 11.6
Hip circumference, cm, mean ± SD	87.9 ± 12.8
Waist hip ratio, mean ± SD	0.9 ± 0.1
Systolic blood pressure, mm Hg, mean ± SD	134.9 ± 22.2
Diastolic blood pressure, mm Hg, mean ± SD	83.5 ± 12.3
Random blood sugar, mg/dl, mm Hg, mean ± SD	152.4 ± 71.2
Behavioural	
Moderate to severe intensity physical activity, n (%;(95% CI))	92 (19.3; 16.0-23.1)
Duration of moderate to severe intensity physical activity, min, n (%;(95% CI))	
<15	324 (67.9; 63.6-71.9)
15-29	61 (12.8; 10.1-16.1)
30-59	75 (15.7; 12.7-19.3)
≥60	17 (3.6; 2.2-5.6)
Current tobacco use status, n (%;(95% CI))	
Nonuser	334 (70.0; 65.7-73.9)
Only Smoker	15 (3.1; 1.9-5.1)
Only Smokeless tobacco user	119 (24.9; 21.3-29.0)
Both smoker and smokeless tobacco user	9 (1.9; 1.0-3.5)
Current alcohol user, n (%;(95% CI))	51 (10.7; 8.2-13.8)
Per capita daily salt intake, gm, median (IQR)	7.1 (4.5; 11.9)
Per capita daily oil intake, ml, median (IQR)	500 (333, 667)
Fruit and vegetable servings taken per day, mean ± SD	3.3 ± 1.0
Co-morbidity	
Co-morbidity status, n (%;(95% CI))	
None	226 (47.4; 42.9-51.8)
Only Hypertension	156 (32.7; 28.6-37.0)
Only Diabetes	49 (10.3; 7.8-13.3)
Both Hypertension and Diabetes	46 (9.6; 7.3-12.6)

**Figure 5:**
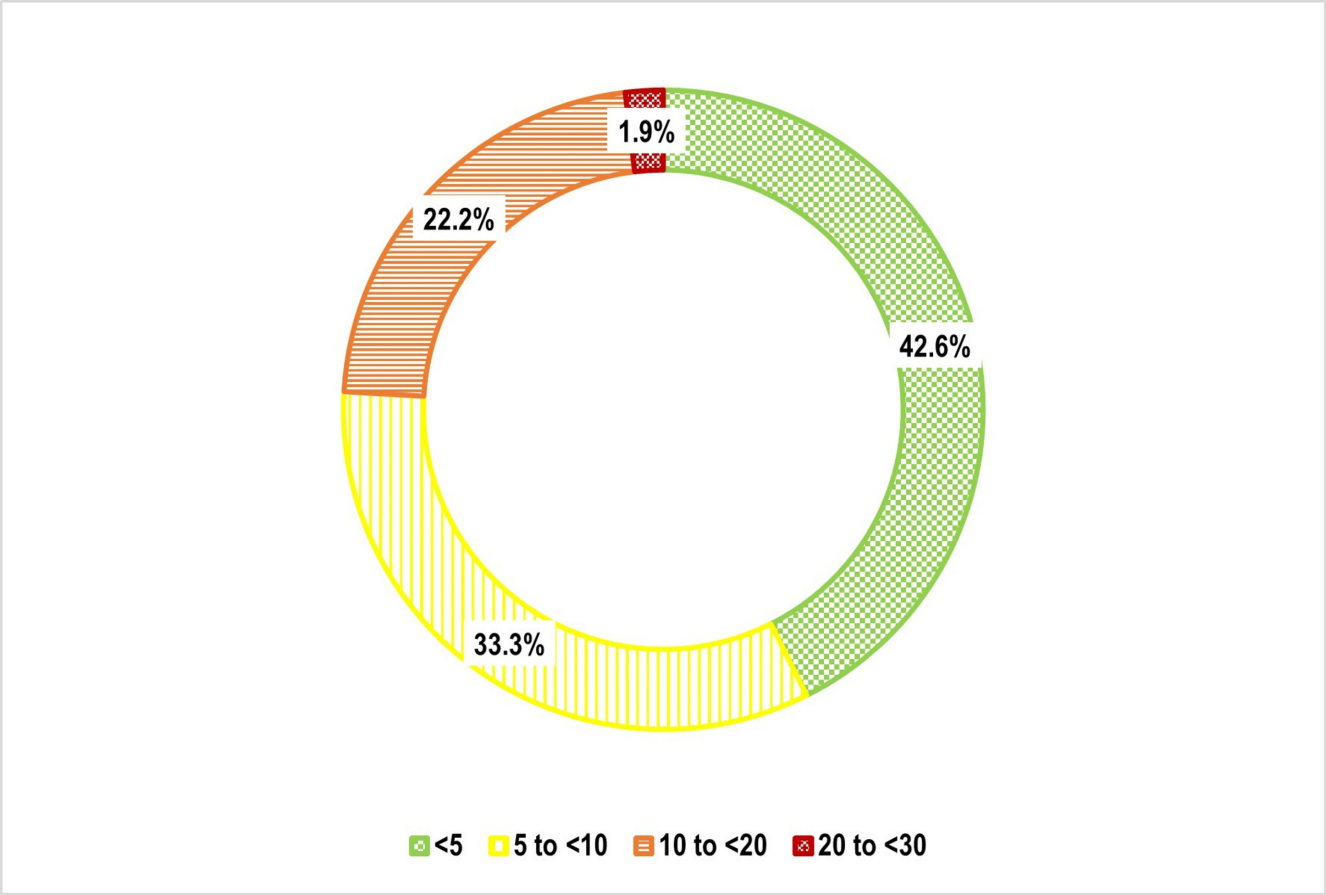
Doughnut diagram showing distribution of the study participants as per their cardiovascular risk: (n=477)

**Figure 6:**
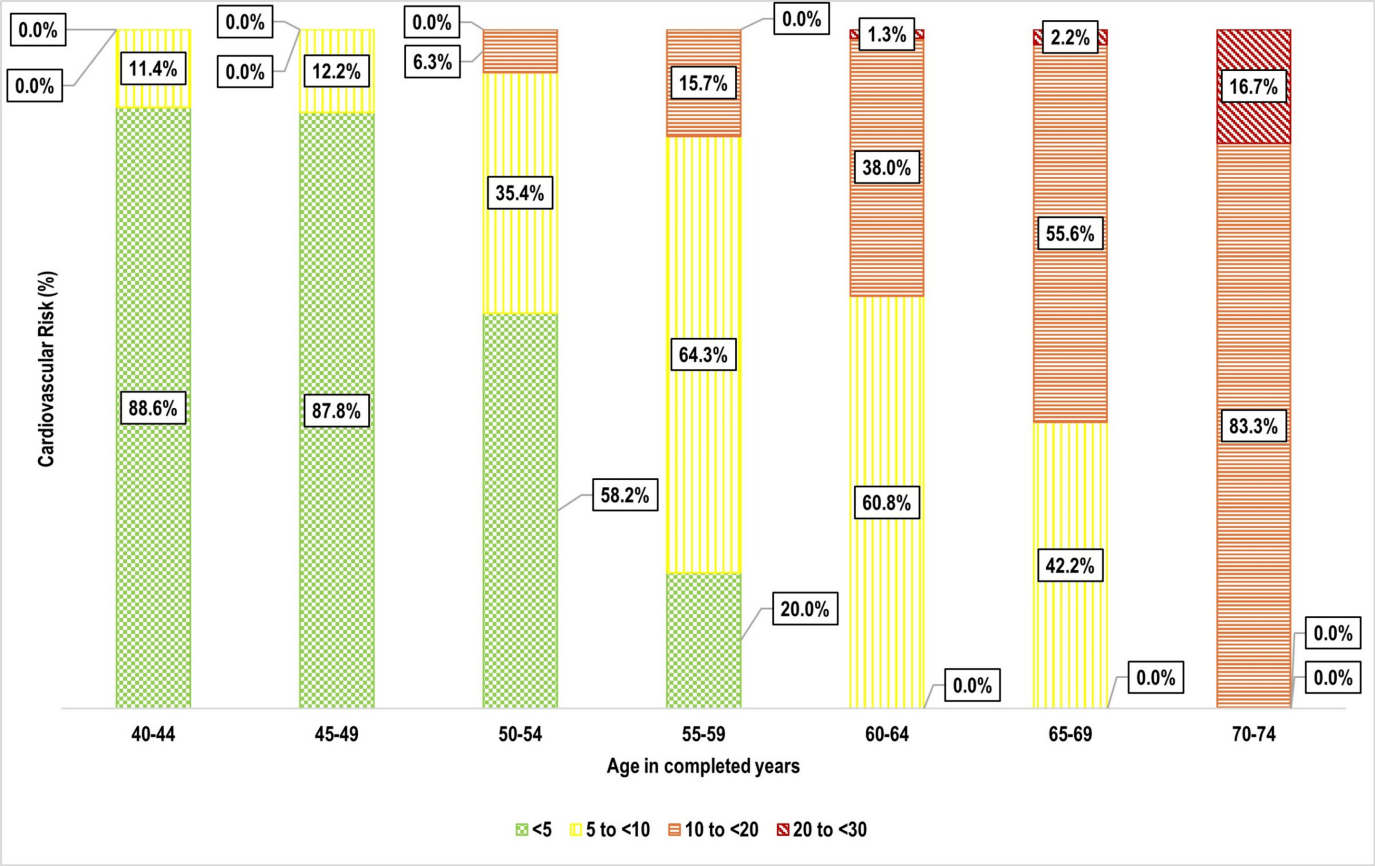
Bar diagram showing distribution of the study participants as per their age and cardiovascular risk: (n=477)

**Figure 7:**
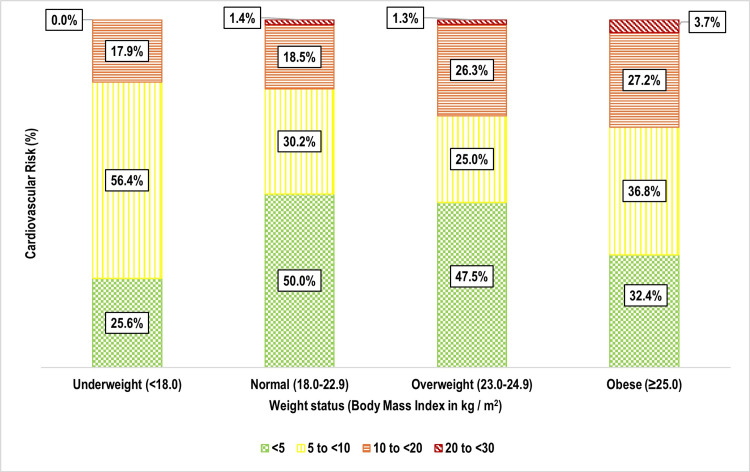
Bar diagram showing distribution of the study participants as per their weight status and cardiovascular risk: (n=477)

**Figure 8:**
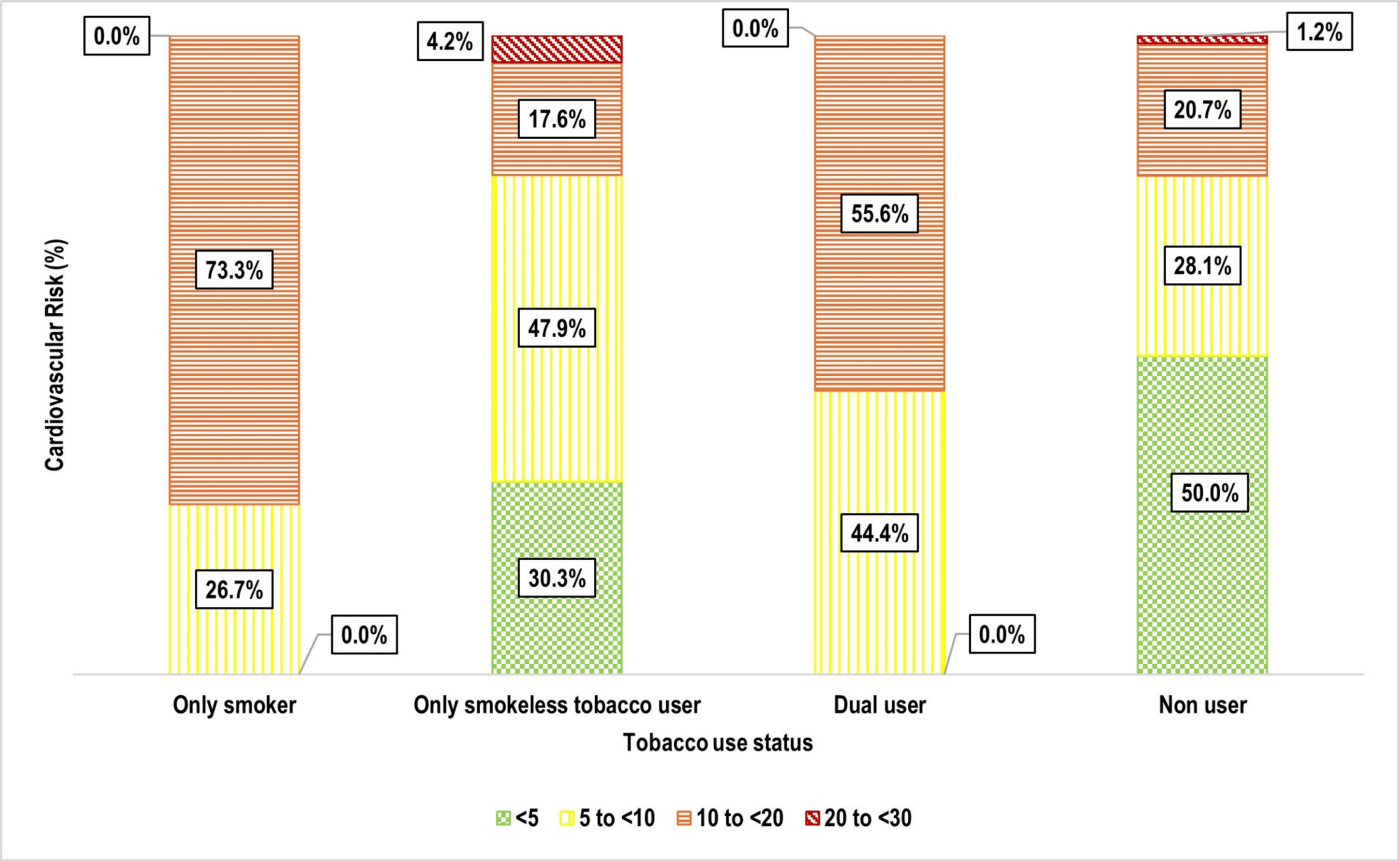
Bar diagram showing distribution of the study participants as per their tobacco use status and cardiovascular risk: (n=477)

**Figure 9:**
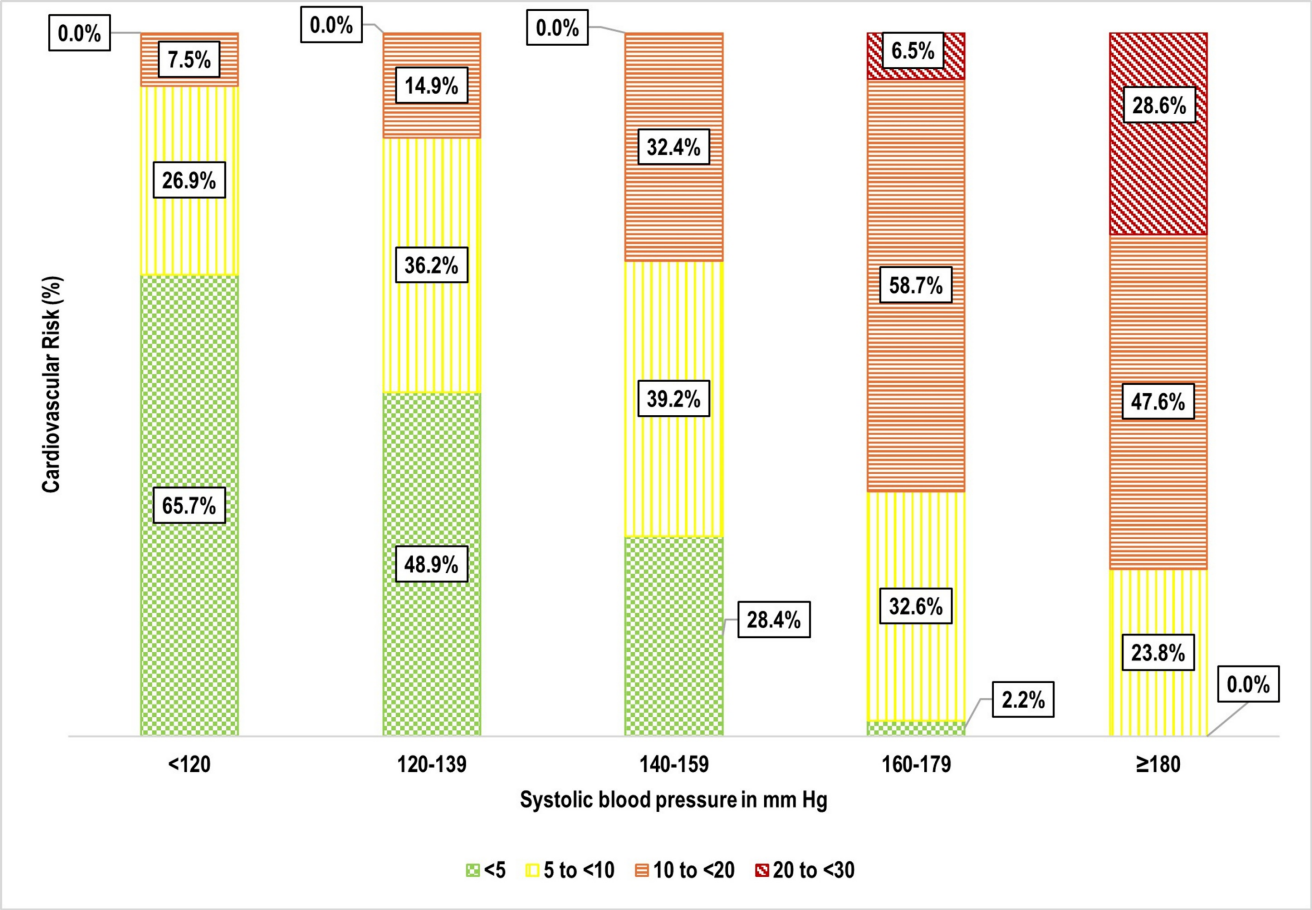
Bar diagram showing distribution of the study participants as per their systolic blood pressure levels and cardiovascular risk: (n=477)

**Figure 10:**
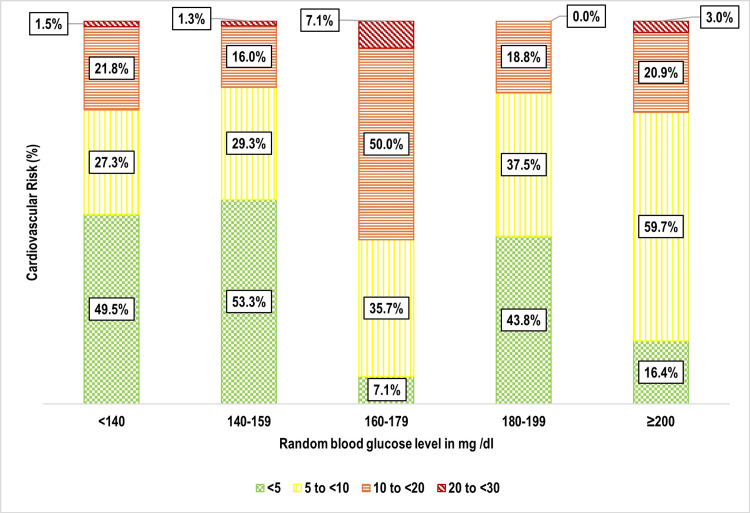
Bar diagram showing distribution of the study participants as per their random blood sugar levels and cardiovascular risk: (n=477)

**Figure 11:**
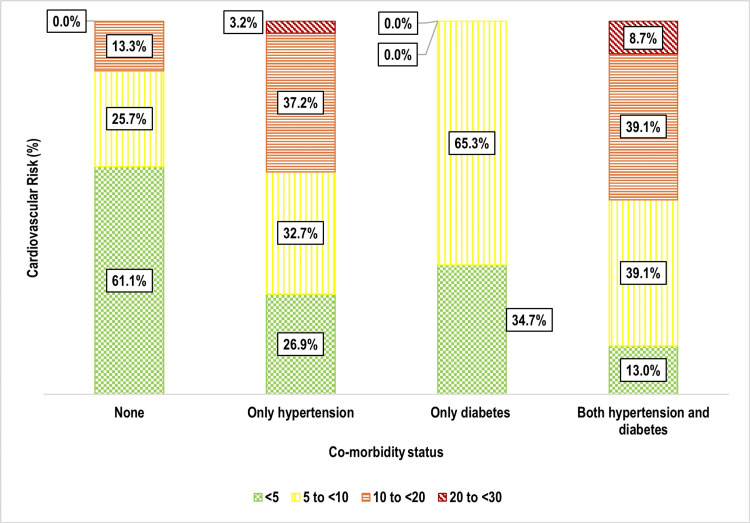
Bar diagram showing distribution of the study participants as per their co-morbidity status and cardiovascular risk: (n=477)

One-third of participants 159 (33.3%) had a 10-year cardiovascular risk of 5% to <10%, followed by 10% to <20% 106 (22.2%), and 20% to <30% nine (1.9%) (Figure 5). Age was strongly associated with cardiovascular risk (p < 0.001), with older individuals exhibiting significantly higher risk levels (Figure 6). Weight status also showed a significant correlation (p = 0.002), with overweight and obese individuals at greater risk (Figure 7). Tobacco use emerged as another key factor (p < 0.001), with smokers and dual users demonstrating significantly higher cardiovascular risk compared to non-users (Figure 8). Systolic blood pressure (SBP) had a strong association with risk (p < 0.001), with SBP levels ≥180 mmHg linked to the highest risk (Figure 9). Similarly, random blood sugar (RBS) levels ≥160 mg/dL were associated with elevated risk (p < 0.001) (Figure 10). Co-morbidity status significantly influenced cardiovascular risk (p < 0.001). The highest risk was observed among individuals with both hypertension and diabetes, followed by those with only hypertension, and then those with only diabetes (Figure 11).

Bivariate multinomial logistic regression analysis identified several factors significantly associated with higher cardiovascular risk, including increasing age, male gender, rural residence, employment, lower per capita monthly income (PCMI), family history of hypertension, diabetes, or heart disease, obesity (BMI ≥ 25 kg/m²), central obesity, and high waist-hip ratio. Lifestyle factors such as tobacco use, alcohol consumption, daily salt intake ≥5 grams, and monthly oil intake ≥500 millilitres also increased the risk. Protective factors included belonging to the Scheduled Caste (SC) or Scheduled Tribe (ST) community, engaging in ≥30 minutes of moderate-to-severe physical activity daily, and consuming ≥5 servings of fruits and vegetables per day. The multinomial logistic regression model identified increasing age, male gender, lower PCMI, family history of hypertension, diabetes, or heart disease, central obesity, and tobacco use as significant risk factors, while regular physical activity was protective. The model explained 81.8% of the variability in cardiovascular risk outcomes (Table 2).

**Table 2:** Bivariate and multivariable multinomial logistic regression analysis identifying predictors of cardiovascular risk among the study participants: (n=477) AOR: adjusted odds ratio, APL: above poverty line, BMI: body mass index,  BPL: below poverty line, CI: confidence interval, kg: kilogram, m: meter, OR: odds ratio, PCMI: per capita monthly income, SC: scheduled caste, ST: scheduled tribe, USD: United States Dollar.

		Cardiovascular Risk (%)					
Variable	Total	5 to <10			≥10		
	N	%	OR (95% CI)	AOR (95% CI)	%	OR (95% CI)	AOR (95% CI)
Sociodemographic Risk Factors							
Age in completed years:	477	33.3	1.4 (1.3-1.4)	1.5 (1.4-1.7)	24.1	1.7 (1.6-1.8)	2.0 (1.8-2.3)
Gender:							
Male	177	42.4	3.9 (2.4-6.2)	10.7 (2.1-53.8)	36.2	5.4 (3.3-9.1)	16.0 (2.4-106.3)
Female	300	28.0	Ref.	Ref.	17.0	Ref.	Ref.
Religion:							
Hindu	454	33.9	1.9 (0.7-5.6)	-	24.0	1.1 (0.4-3.1)	-
Muslim	23	21.7	Ref.		26.1	Ref.	
Caste:							
SC/ST	167	40.7	1.2 (0.8-1.9)	1.4 (0.6-3.3)	13.2	0.4 (0.2-0.7)	0.4 (0.1-1.2)
Others	310	29.4	Ref.	Ref.	30.0	Ref.	Ref.
Marital Status:							
Currently married	451	33.5	0.9 (0.3-2.3)	-	23.5	0.5 (0.2-1.4)	-
Others	26	30.8	Ref.		34.6	Ref.	
Educational Level:							
Secondary and above	99	38.4	1.1 (0.7-1.8)	2.6 (0.8-8.2)	16.2	0.6 (0.3-1.0)	1.4 (0.3-6.5)
Below Secondary	378	32.0	Ref.	Ref.	26.2	Ref.	Ref.
Place of residence:							
Rural	257	37.7	2.2 (1.4-3.3)	1.5 (0.5-5.0)	29.2	2.6 (1.6-4.2)	0.9 (0.2-4.3)
Urban	220	28.2	Ref.	Ref.	18.2	Ref.	Ref.
Socioeconomic Risk Factors							
Work for pay:							
Yes	256	38.7	3.1 (1.9-4.7)	0.3 (0.1-1.1)	33.6	5.5 (3.3-9.2)	0.4 (0.1-1.8)
No	221	27.1	Ref.	Ref.	13.1	Ref.	Ref.
PCMI in USD:							
<21.8	121	43.8	1.9 (1.2-3.2)	3.0 (1.0-8.9)	22.3	1.2 (0.7-2.1)	3.7 (0.9-13.8)
≥21.8	356	29.8	Ref.	Ref.	24.7	Ref.	Ref.
Socio-economic status:							
APL	365	32.1	0.7 (0.4-1.2)	-	23.8	0.8 (0.5-1.4)	-
BPL	112	37.5	Ref.		25.0	Ref.	
Family History related Risk Factors							
Family history of high blood pressure, diabetes and heart disease:							
Yes	121	39.7	2.0 (1.2-3.3)	3.6 (1.5-8.7)	30.6	2.2 (1.3-3.7)	5.7 (1.8-18.4)
No	356	31.2	Ref.	Ref.	21.9	Ref.	Ref.
Anthropometric Risk Factors							
Obese (BMI in kg/m^2^):							
Yes (≥ 25)	136	36.8	1.6 (1.0-2.7)	1.1 (0.4-2.8)	30.9	2.1 (1.3-3.5)	1.4 (0.4-4.7)
No (< 25)	341	32.0	Ref.	Ref.	21.4	Ref.	Ref.
Centrally Obese:							
Yes	211	36.0	1.8 (1.2-2.8)	4.7 (1.7-13.0)	31.8	2.8 (1.7-4.4)	11.9 (3.5-40.9)
No	266	31.2	Ref.	Ref.	18.0	Ref.	Ref.
High Waist Hip Ratio:							
Yes	356	34.8	1.9 (1.2-3.1)	0.3 (0.1-1.1)	28.1	3.6 (1.9-6.6)	0.8 (0.2-4.0)
No	121	28.9	Ref.	Ref.	12.4	Ref.	Ref.
Behavioural Risk Factors							
Physically active:							
Yes	92	27.2	0.5 (0.3-0.9)	0.4 (0.1-0.9)	14.1	0.3 (0.2-0.7)	0.2 (0.1-0.8)
No	385	34.8	Ref.	Ref.	26.5	Ref.	Ref.
Tobacco User:							
Yes	143	45.5	3.2 (1.9-5.2)	5.2 (1.8-14.9)	29.4	2.7 (1.6-4.5)	8.2 (2.0-33.6)
No	334	28.1	Ref.	Ref.	21.9	Ref.	Ref.
Alcohol User:							
Yes	51	51.0	3.4 (1.6-7.1)	2.0 (0.5-8.1)	27.5	2.4 (1.1-5.5)	2.1 (0.4-12.4)
No	426	31.2	Ref.	Ref.	23.7	Ref.	Ref.
Per capita daily salt intake in grams:							
≥5	330	35.2	2.1 (1.3-3.3)	1.4 (0.4-4.6)	30.3	5.2 (2.8-9.6)	3.9 (0.8-20.2)
<5	147	29.3	Ref.	Ref.	10.2	Ref.	Ref.
Addition of extra salt while having food:							
Yes	169	38.5	1.4 (0.9-2.2)	0.6 (0.2-2.3)	22.5	1.0 (0.6-1.7)	0.3 (0.1-1.6)
No	308	30.5	Ref.	Ref.	25.0	Ref.	Ref.
Consumes salt rich food:							
Yes	136	39.0	1.5 (0.9-2.4)	0.8 (0.2-2.7)	24.3	1.2 (0.7-2.1)	0.8 (0.2-4.2)
No	341	31.1	Ref.	Ref.	24.0	Ref.	Ref.
Per capita monthly Oil intake in millilitres:							
≥500	276	37.3	2.3 (1.5-3.5)	2.6 (0.9-6.8)	30.1	3.2 (1.9-5.3)	1.6 (0.4-5.6)
<500	201	27.9	Ref.	Ref.	15.9	Ref.	Ref.
Fruit and vegetable servings taken per day:							
≥5	61	24.6	0.4 (0.2-0.8)	0.6 (0.2-2.2)	9.8	0.2 (0.1-0.5)	0.3 (0.1-1.6)
<5	416	34.6	Ref.	Ref.	26.2	Ref.	Ref.

## Discussion

This community-based cross-sectional study assessed cardiovascular risk factors and their predictors in a low-resource setting. About one-third of participants had low cardiovascular risk, while nearly one-fourth had moderate to high risk. Central obesity was observed in over half of the individuals, and one-third were tobacco users, predominantly smokeless forms. Hypertension affected one in three participants, while one in five had diabetes. Physical inactivity was highly prevalent, with nearly three-fourths of participants not engaging in regular exercise. Increasing age, male gender, obesity, tobacco use, and family history of chronic conditions were significant predictors, while regular physical activity and adequate fruit and vegetable intake were protective factors.

The present study, conducted among a population with nearly equal rural and urban representation in Deoghar, Jharkhand, revealed that 75.8% of participants had low cardiovascular risk (<10%), 22.2% had moderate risk (10% to <20%), and 1.9% had high risk (≥20%). These findings align closely with Deori et al. [16], who reported 76.8% low risk, 12.8% moderate risk, and 10.4% high risk in a rural population in Lucknow. Similarly, Mohamed et al. [27], studying outpatients (OPD) in Puducherry, found 76.9% low risk, 15.9% moderate risk, and 7.0% high risk. Ghorpade et al. [15], examining a rural South Indian population, observed 79.2% low risk, 8.3% moderate risk, and 12.5% high risk. Amoghashree et al. [14], studying tribal populations in Karnataka, reported slightly lower moderate and high risks, with 83.0% low risk, 6.8% moderate risk, and 10.2% high risk. In contrast, Bansal et al. [28], analysing patients attending a Rural Health Training Centre (RHTC) in Punjab, reported only 56.0% low risk, with 44.0% in the combined moderate-to-high risk categories. At a national level, Kulothungan et al. [17], using National Noncommunicable Disease Monitoring Survey (NNMS) data, found a higher proportion of low-risk individuals (84.9%), with 14.4% moderate risk and 0.7% high risk. Similarly, Mamgai et al. [29], analysing Longitudinal Ageing Study in India (LASI) data, reported 68.8% low risk, 28.4% moderate risk, and 2.8% high risk, indicating a slightly higher moderate-risk prevalence compared to our study.

International comparisons reveal further variation. Khanal et al. [30], studying a community-based population in Nepal, observed a much higher proportion of low risk (86.4%), with 9.3% moderate risk and 4.3% high risk. Babatunde et al. [31], analysing Nigerian civil servants, reported a comparable 76.9% low risk, 8.5% moderate risk, and 2.8% high risk. Rezaei et al. [32], examining a population-based sample in Iran, found 76.1% low risk, 18.2% moderate risk, and 5.7% high risk, reflecting slightly higher moderate-to-high risk profiles than in our findings. These variations across studies reflect differences in population characteristics, study settings, sampling methods, and rural-urban compositions. Methodological differences in cardiovascular risk assessment and the prevalence of risk factors such as obesity, hypertension, diabetes, tobacco use, and physical inactivity further contribute to these disparities. Cultural, dietary, socioeconomic, and healthcare access disparities across regions also play a critical role in shaping cardiovascular risk profiles, emphasizing the importance of localized interventions and context-specific strategies to address cardiovascular health.

In the present study, cardiovascular risk increased twofold with each unit increase in age, consistent with findings from Indian studies by Amoghashree et al. [14], Mohamed et al. [27], Deori et al. [16], and Bansal et al. [28], as well as international studies by Khanal et al. [30] and Babatunde et al. [31]. This might be due to the cumulative impact of age-related physiological changes, such as arterial stiffening, increased oxidative stress, and metabolic decline, which elevate the risk for conditions like hypertension and diabetes. Males had 16 times higher odds for moderate-to-high cardiovascular risk compared to females, similar to findings by Deori et al. [16] and Mohamed et al. [27]. This might be due to a higher prevalence of behavioural risk factors among men, such as tobacco and alcohol use, as well as differences in health-seeking behavior and hormonal protection in premenopausal women. Scheduled Caste (SC) and Scheduled Tribe (ST) participants had 60% lower odds for moderate-to-high cardiovascular risk in the present study. This might be due to differences in traditional dietary practices, physical activity levels, or other sociocultural factors that provide a protective effect against cardiovascular risk.

Participants residing in rural areas had 2.6 times higher odds for moderate-to-high cardiovascular risk, contrasting with findings by Kulothungan et al. [17], where urban residents had 1.3 times higher odds. This might be due to differences in healthcare access, awareness, and dietary practices between rural and urban populations. Rural residents might face limited access to preventive healthcare and unhealthy dietary patterns, while urban residents often have higher exposure to sedentary lifestyles and stress. Employed individuals in this study had 5.5 times higher odds for moderate-to-high cardiovascular risk, differing from findings by Amoghashree et al. [14], where unemployed individuals had higher risk, and Khanal et al. [30], where unemployed or retired individuals were at higher risk. This might reflect occupational stress, irregular work hours, or limited time for physical activity among employed individuals. Lower PCMI emerged as a significant predictor of cardiovascular risk, consistent with findings by Mamgai et al. [29] and Balaji et al. [33]. This might be due to financial constraints limiting access to nutritious food, preventive healthcare, and effective chronic disease management. A family history of hypertension, diabetes, or heart disease was associated with 5.7 times higher odds for moderate-to-high cardiovascular risk in this study. This might be due to genetic predisposition and shared environmental and lifestyle factors within families, which can increase susceptibility to cardiovascular conditions.

Obesity was associated with 2.1 times higher odds for moderate-to-high cardiovascular risk, consistent with findings by Amoghashree et al. [14], Deori et al. [16], and Babatunde et al. [31]. Central obesity was a stronger predictor, with 11.9 times higher odds, similar to findings by Balaji et al. [33], Babatunde et al. [31], and Kulothungan et al. [17] (1.4 times higher odds). This might be due to the metabolic effects of central obesity, including insulin resistance, inflammation, and dyslipidaemia, which are key contributors to cardiovascular risk. Tobacco use was associated with 8.2 times higher odds for moderate-to-high cardiovascular risk, consistent with findings by Amoghashree et al. [14], Deori et al. [16], Balaji et al. [33], and Mohamed et al. [26]. This might be due to the damaging effects of tobacco on vascular health, including endothelial dysfunction and arterial stiffness. Alcohol use was associated with 2.4 times higher odds, similar to findings by Deori et al. [16]. This might reflect the adverse impact of alcohol on blood pressure and lipid metabolism.

Excessive daily salt intake (≥5 grams) was associated with 5.2 times higher odds for moderate-to-high cardiovascular risk, while monthly oil consumption (≥500 milliliters) was associated with 3.2 times higher odds. These dietary habits might contribute to hypertension and obesity, both of which are significant cardiovascular risk factors. Protective factors included consuming ≥5 servings of fruits and vegetables daily, which reduced cardiovascular risk by 80%, and engaging in at least 30 minutes of moderate-to-severe physical activity, which also reduced risk by 80%. These findings align with those of Babatunde et al. [31], Kulothungan et al. [17], and Mamgai et al. [29]. Kulothungan et al. [17] reported that insufficient physical activity increased cardiovascular risk by 1.6 times, while Mamgai et al. [29] found regular exercise reduced risk by 32%. The protective effects might be due to the role of fruits and vegetables in improving antioxidant levels and reducing inflammation, while physical activity improves cardiovascular function and metabolic health.

The INTERHEART study [34], a landmark global case-control study, identified 100 key modifiable risk factors - spanning lifestyle, metabolic, and social determinants - that account for over 90% of myocardial infarction risk. Many of these were prominent in our study population. Among lifestyle factors, tobacco use significantly increased cardiovascular risk, while physical inactivity was widespread, reinforcing the protective role of regular physical activity. Alcohol use was also associated with higher cardiovascular risk, with country liquor being the most commonly consumed form. Although unhealthy diet was not directly quantified, low fruit and vegetable intake, high salt consumption, and excessive oil intake indicate a substantial dietary risk burden. Among metabolic factors, central obesity strongly predicted increased cardiovascular risk, alongside hypertension and diabetes. However, unlike the INTERHEART study, abnormal lipid levels (apolipoprotein B/apolipoprotein A1 (ApoB/ApoA1)) were not assessed due to the non-laboratory-based approach of our study. While psychosocial stress and depression were not directly measured, lower income was associated with higher cardiovascular risk, potentially reflecting financial stress and limited access to healthcare.

This study highlights opportunities to reduce cardiovascular risk in low-resource settings by addressing modifiable factors such as central obesity, tobacco use, physical inactivity, and inadequate fruit and vegetable intake. Protective behaviors, including regular physical activity and sufficient dietary intake, were associated with lower cardiovascular risk, but the dynamics of these behaviors, including their adoption, barriers, and sustainability, warrant further evaluation. The higher risk observed among males, rural residents, and individuals with lower socioeconomic status underscores the need for targeted, context-specific interventions. Additionally, regional factors such as dietary patterns, cultural practices, and physical activity levels warrant further investigation to understand their influence on cardiovascular risk.

The study had some limitations. Firstly, its cross-sectional design limits the ability to establish causal relationships between cardiovascular risk factors and outcomes. Secondly, reliance on self-reported data for behaviors such as tobacco use, physical activity, and dietary habits may have introduced recall and social desirability biases, potentially affecting accuracy. Thirdly, while the non-laboratory-based WHO-ISH risk prediction charts were resource-efficient for low-resource settings, they do not account for cholesterol levels, restricting the assessment of cardiovascular risk due to hypercholesterolemia. Measuring cholesterol requires a fasting blood sample, which was not feasible in this community-based study with limited resources. However, prior Indian [13,35] and international [32,36,37] studies have shown good agreement between non-laboratory-based and laboratory-based risk charts, supporting their validity. Additionally, psychosocial stress and depression - recognized traditional cardiovascular risk factors in the INTERHEART study [34] - were not assessed due to feasibility constraints. Furthermore, alternative cardiovascular risk assessment models such as QResearch (cardiovascular risk algorithm) estimated version 3 (QRISK3), Framingham Risk Score for Coronary Heart Disease (FRS-CHD), American College of Cardiology/American Heart Association (ACC/AHA)-Atherosclerotic Cardiovascular Disease (ASCVD) SCORE, Global Registry of Acute Coronary Events (GRACE) risk score, and Selecting Patients Of Rheumatic Heart Disease Undergoing Valve Surgery For Pre-Surgical Coronary Angiography (SERENE-CAG) risk score, which have been validated for the Indian population [38], were not included due to their reliance on laboratory-based parameters such as lipid profile, creatinine, and cardiac enzymes, which were impractical to assess in this resource-limited, community-based setting. Lastly, the study’s focus on specific rural and urban outreach areas in Deoghar, combined with convenience sampling and voluntary participation, may have introduced selection bias, limiting the generalizability of findings to other populations.

## Conclusions

In this community-based study from Eastern India, approximately one in four adults aged 40-74 years had a moderate-to-high 10-year cardiovascular risk as per the WHO/ISH non-laboratory chart. Central obesity and tobacco use were the strongest independent predictors of elevated risk, while increasing age, male gender, lower per capita income, and a positive family history of hypertension, diabetes, or heart disease also significantly contributed. Regular physical activity was found to be a strong protective factor. The findings highlight the urgent need for targeted interventions addressing obesity, tobacco use, and physical inactivity to reduce cardiovascular risk among older adults in similar low-resource settings.
